# High Flow Scalp Arteriovenous Malformation: A Case Report

**DOI:** 10.29252/wjps.9.2.234

**Published:** 2020-05

**Authors:** Hamed Ghoddusi Johari, Reza Shahriarirad, Amirhossein Erfani, Mohammad Hasan Darabi

**Affiliations:** 1 Thoracic and Vascular Surgery Research Center, Shiraz University of Medical Sciences, Shiraz, Iran;; 2 Research Center for Bone and Joint Diseases, Department of Orthopedic Surgery, Chamran Hospital, Shiraz University of Medical Sciences, Shiraz, Iran;; 3 Student Research Committee, Shiraz University of Medical Sciences, Shiraz, Iran

**Keywords:** Arteriovenous malformation, Scalp, Surgery, Vascular

## Abstract

Scalp arteriovenous malformations (AVMs) are abnormal vascular lesions, which can be managed effectively and safely with surgical or endovascular approaches. Here, we discuss a case of scalp AVM malformation in a 25-year-old female, in which due to the proximity of the feeder artery to right orbit, surgical excision was preferred and the AVM was excised with an uneventful post-op course.

## INTRODUCTION

Scalp arteriovenous malformations (AVMs) are relatively rare and can be either traumatic or spontaneous. These lesions may be found incidentally or owing to signs and symptoms that they produce such as headache, epilepsy, tinnitus, hemorrhage and focal neurological deficit.^1^ These lesions often constitute high-flow arterial blood from the superficial temporal or occipital arteries with venous outflow into extracranial venous structures. Elimination of bothersome symptoms like headaches and tinnitus along with cosmetic relief of the visible mass are the main indications of treatment.^1^ Here, we present a case of scalp AVM in a young woman who underwent successful surgical excision of AVM.

## CASE REPORT

A 25 y/o female complained from a pulsatile mass over her right frontal area. She had no history of trauma. Physical examinations revealed a large pulsatile mass-like lesion measuring about 5x5x2 cm with at least 2 feeder arteries on the scalp and one feeder artery from the right superficial temporal artery. The involved scalp was tender. There was no evidence of any facial nerve compromise or any other neurologic deficits. Brain and neck CT angiography was performed to rule out any intracranial or extracranial vascular lesions. 

**Fig. 1:**
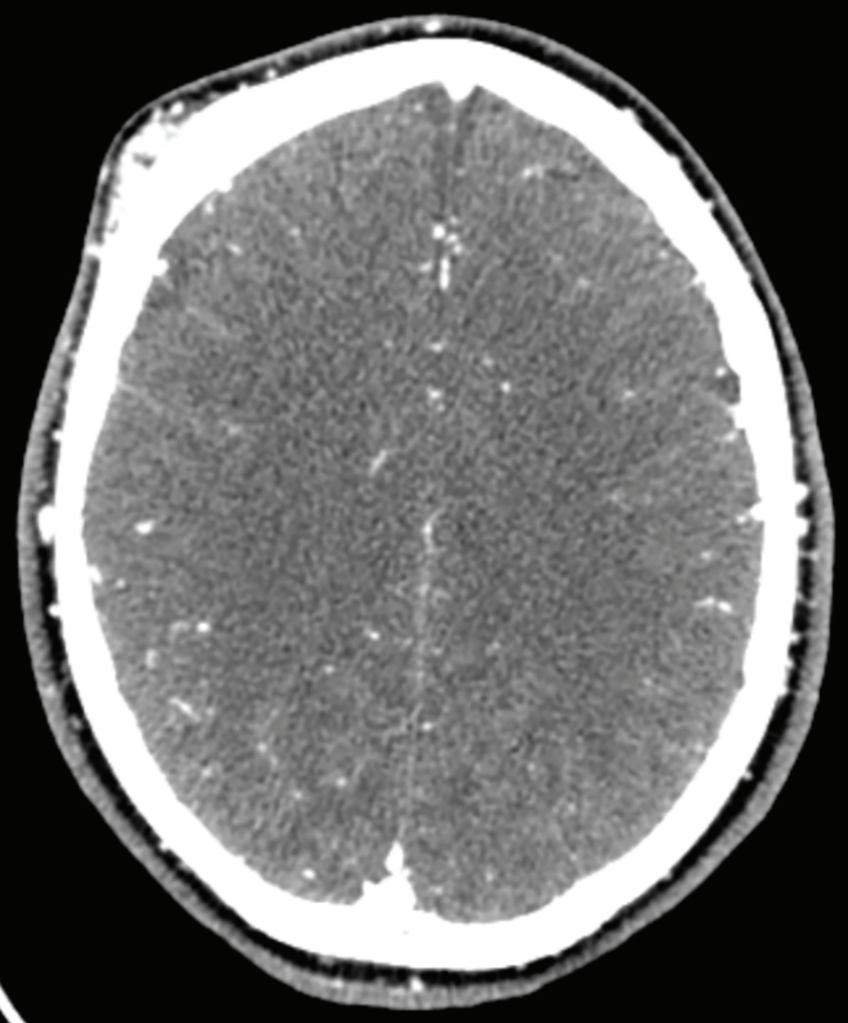
Brain CT-scan demonstrating arteriovenous malformation on the scalp

**Fig. 2:**
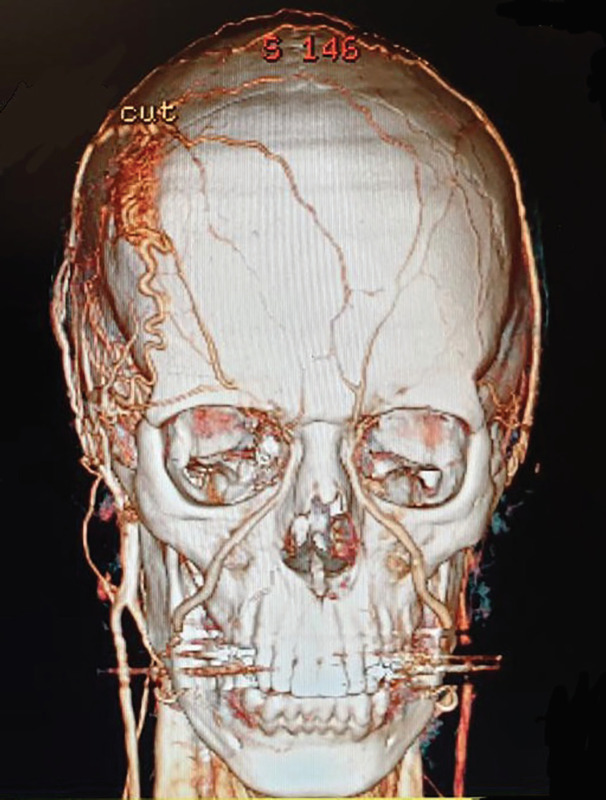
CT angiography revealing arteriovenous malformation in the scalp

Spiral CT-angiography of cervical region demonstrated multiple dilated tortuous vessels in subcutaneous fat of right side of the scalp in the right frontal and supplied by branching from right external carotid artery suggestive of scalp arteriovenous malformation in the right frontal area. There were no signs of any intracranial involvement or bone erosion (Figures 1 and 2). Also, spiral CT angiography of the brain was normal with no sign of AVM. 

**Fig. 3:**
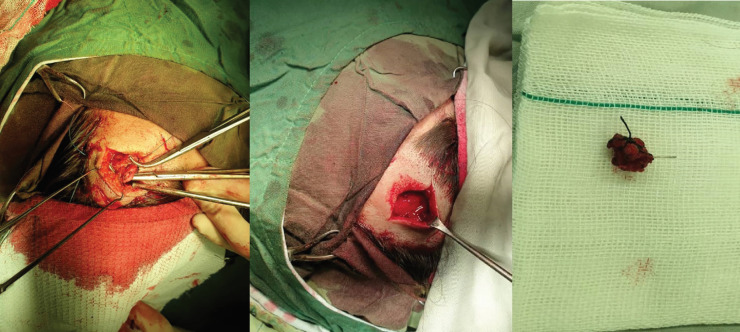
Surgical resection of arteriovenous malformations (AVM) of the scalp

Due to the proximity of one feeder artery to the right orbit, we decided to choose a surgical approach instead of the endovascular approach to prevent any ophthalmic complications. A written consent was provided from the patient. First, via two facial incisions, superficial temporal artery was ligated and then, circumferential sutures were applied on the scalp for transient bleeding control, and all feeding arteries were ligated and AVM was resected completely (Figure 3). Finally, hemostatic sutures were removed and the patient had an uneventful post-op course.

## DISCUSSION

The first report of an AVM was in 1757 by Wilham Hunter.^2^ After that, numerous terms including aneurysmal serpentinum, aneurysm cirsoides, plexiform angioma, arteriovenous fistula, and AVM were used to label the vascular malformations of the scalp.^3^^-^^5^ In clinical practice, these lesions are rather rare, in which the most common sites of involvement in the scalp are usually the temporal, frontal, and parietal regions.^3^^,^^5^^,^^6^

AVMs of the scalp are abnormal connections between superficial scalp veins and arteries. Although any scalp vessel may be involved, they are regularly fed by occipital arteries and/or superficial temporal arteries.^7^^-^^10^ Clinical presentations are usually related to the size of the AVM, in which some patients may present with numbness, headache, and/or hemorrhage, while others may present with severe symptoms such as scalp lesions. The etiology of scalp AVMs may be either traumatic or spontaneous, which mostly develops in patients over 30 years old and in the trauma background.^4^^,^^11^

However, spontaneous AVM of the scalp can be present at birth but is asymptomatic until adulthood in most patients.^3^^,^^11^^,^^12^ Deterioration of the symptoms in these cases is usually caused due to trauma, pregnancy, or hormonal changes. Most patients regularly pursue medical care in their second decade.^3^^,^^11^^,^^12^ In our case, there was no history or evidence of trauma or fracture, and also the symptoms were presented at 24 years old. Hence, the case was seemingly a spontaneous AVM.

Scalp AVMs are most frequently confused with hemangioma and cavernomas, and they are usually seen as well-demarcated lesions. For the surgical technique to be performed, the definite diagnosis is essential, and cranial angiography is of extreme importance for diagnosis and treatment selection; Therefore, the preoperative radiological evaluation should be used for the determination of feeding arteries, drainage vessels, connected vascular structures, numbers of fistulas, and shunt flow volume to prevent any potential complications.^3^^,^^5^^,^^13^

Relieving the clinical complaints disturbing the patient’s comfort is the main goal of scalp AVM treatment. Numerous therapeutic modalities have been described to manage these lesions, such as surgical excision, transarterial and transvenous embolization, ligation of feeding vessels, injection of sclerosing agents into the nidus, and electro thrombosis.^14^ Surgical excision is particularly effective in AVMs and is the most commonly used treatment method.^4^^,^^13^^,^^15^ Due to the abnormal arteriovenous communication, AVM must be eliminated because recurrence or enlargement is described after an incomplete treatment.^16 ^

The most essential step is total surgical excision without triggering scalp necrosis and excessive blood loss. Additionally, in this way, a better cosmetic result may be obtained.^3^^,^^17^^,^^18^ Hemorrhage is one of the most important complications throughout the operation, in which various techniques including percutaneous sutures of the feeding vessels, interlocking suture along the line of incision and use of a tourniquet and intestinal clamp over the base of the flap, have been used to control the hemorrhage throughout the surgery.^3^^,^^14^

Endovascular treatment may be useful to reduce the hemorrhage and assist the surgical management or in the direct treatment of AVMs.^4^^,^^13^^,^^15^ Preoperative embolization of nidus and feeders prevents especially the massive hemorrhages. In the case of treatment of large scalp AVM, embolization and endovascular treatment may not be sufficient. Management of an AVM with embolization may be accompanied by risks of embolization of non-target arteries, local inflammation, and soft tissue tattooing. It is also associated with a higher rate of recurrence compared with surgical excision.^19^^,^^20^

In our case, the decision was made to avoid endovascular embolization and to prevent ophthalmic complications. Instead, the surgical excision of the AVM was more simple and safe. So an understanding of the arterial supply of scalp AVM is very important in making a therapeutic decision and a surgical approach is very safe and effective in highly selected cases. 

## References

[1] Fakharian E, Baboly AG, Yazdipur P (2018). Traumatic arteriovenous malformation of scalp: A case report. Archives of Trauma Research¦ Volume.

[2] Gurkanlar D, Gonul M, Solmaz I, Gonul E (2006). Cirsoid aneurysms of the scalp. Neurosurg Rev.

[3] Shenoy SN, Raja A (2004). Scalp arteriovenous malformations. Neurol India.

[4] Massimi L, De Bonis P, Esposito G, Novegno F, Pettorini B, Tamburrini G, Caldarelli M, Di Rocco C (2009). Vertex scalp mass as presenting sign of a complex intracranial vascular malformation. J Neurosurg Pediatr.

[5] Heilman CB, Kwan ES, Klucznik RP, Cohen AR (1990). Elimination of a cirsoid aneurysm of the scalp by direct percutaneous embolization with thrombogenic coils. Case report. J Neurosurg.

[6] Burrus TM, Miller GM, Flynn LP, Fulgham JR, Lanzino G (2009). NeuroImages. Symptomatic left temporal arteriovenous traumatic fistula. Neurology.

[7] Schultz RC, Hermosillo CX (1980). Congenital arteriovenous malformation of the face and scalp. Plast Reconstr Surg.

[8] Richter GT, Suen JY (2011). Pediatric extracranial arteriovenous malformations. Curr Opin Otolaryngol Head Neck Surg.

[9] Visser A, FitzJohn T, Tan ST (2011). Surgical management of arteriovenous malformation. J Plast Reconstr Aesthet Surg.

[10] Rutledge C, Nelson J, Lu A, Nisson P, Jonzzon S, Winkler EA, Cooke D, Abla AA, Lawton MT, Kim H (2020). Cost determinants in management of brain arteriovenous malformations. Acta Neurochir (Wien).

[11] Fisher-Jeffes ND, Domingo Z, Madden M, de Villiers JC (1995). Arteriovenous malformations of the scalp. Neurosurgery.

[12] Li F, Zhu S, Liu Y, Chen Y, Chi L, Chen G, Zhang J, Qu F (2007). Traumatic arteriovenous fistula of the superficial temporal artery. J Clin Neurosci.

[13] Gupta AK, Purkayastha S, Bodhey NK, Kapilamoorthy TR, Krishnamoorthy T, Kesavadas C, Thomas B (2008). Endovascular treatment of scalp cirsoid aneurysms. Neurol India.

[14] Chowdhury FH, Haque MR, Kawsar KA, Sarker MH, Momtazul Haque AF (2013). Surgical management of scalp arterio-venous malformation and scalp venous malformation: An experience of eleven cases. Indian J Plast Surg.

[15] Tiwary SK, Khanna R, Khanna AK (2007). Craniofacial cirsoid aneurysm: 2-stage treatment. J Oral Maxillofac Surg.

[16] Chowdhury FH, Haque MR, Kawsar KA, Sarker MH, Momtazul Haque AF (2013). Surgical management of scalp arterio-venous malformation and scalp venous malformation: An experience of eleven cases. Indian J Plast Surg.

[17] Matsushige T, Kiya K, Satoh H, Mizoue T, Kagawa K, Araki H (2004). Arteriovenous malformation of the scalp: case report and review of the literature. Surg Neurol.

[18] Senoglu M, Yasim A, Gokce M, Senoglu N (2008). Nontraumatic scalp arteriovenous fistula in an adult: technical report on an illustrative case. Surg Neurol.

[19] Arat A, Cil BE, Vargel I, Turkbey B, Canyigit M, Peynircioglu B, Arat YO (2007). Embolization of high-flow craniofacial vascular malformations with onyx. AJNR Am J Neuroradiol.

[20] Liu AS, Mulliken JB, Zurakowski D, Fishman SJ, Greene AK (2010). Extracranial arteriovenous malformations: natural progression and recurrence after treatment. Plast Reconstr Surg.

